# Potential Conservation of Circadian Clock Proteins in the *phylum* Nematoda as Revealed by Bioinformatic Searches

**DOI:** 10.1371/journal.pone.0112871

**Published:** 2014-11-14

**Authors:** Andrés Romanowski, Matías Javier Garavaglia, María Eugenia Goya, Pablo Daniel Ghiringhelli, Diego Andrés Golombek

**Affiliations:** 1 Laboratorio de Cronobiología, Dto de Ciencia y Tecnología, Universidad Nacional de Quilmes, Bernal, Buenos Aires, Argentina; 2 Laboratorio de Ingeniería Genética y Biología Celular y Molecular – Área Virosis de Insectos (LIGBCM – AVI), Instituto de Microbiología Básica y Aplicada (IMBA), Dto de Ciencia y Tecnología, Universidad Nacional de Quilmes, Bernal, Buenos Aires, Argentina; University of Fribourg, Switzerland

## Abstract

Although several circadian rhythms have been described in *C. elegans*, its molecular clock remains elusive. In this work we employed a novel bioinformatic approach, applying probabilistic methodologies, to search for circadian clock proteins of several of the best studied circadian model organisms of different taxa (*Mus musculus, Drosophila melanogaster, Neurospora crassa, Arabidopsis thaliana* and *Synechoccocus elongatus*) in the proteomes of *C. elegans* and other members of the *phylum Nematoda*. With this approach we found that the *Nematoda* contain proteins most related to the core and accessory proteins of the insect and mammalian clocks, which provide new insights into the nematode clock and the evolution of the circadian system.

## Introduction

*C. elegans* is a soil-dwelling nematode subjected to daily changes in environmental variables in its natural habitat. Several daily and circadian rhythms have been described in this organism, including locomotor and swimming activity, tolerance to abiotic and biotic stress, metabolic variables and melatonin synthesis [1]–[6]. However, little is known about the molecular mechanism that governs its circadian behaviors, in comparison to information related to other model organisms. In particular, early studies in the fruit fly *Drosophila melanogaster* suggested a feedback mechanism involving a transcription-translation oscillator (TTO), based on the observation that the oscillation of the *period* gene at the mRNA and protein level, as well as their interaction, was required to maintain rhythmicity [7]. However, it was not clear whether proteins exerted a direct effect over the mRNA or acted by means of other biochemical signals. This was later complemented with studies in the fungus *Neurospora crassa*, which showed that the FREQUENCY (FRQ) protein was capable of regulating its own transcription, by means of a negative feedback loop [8]. At the present time, the transcriptional-translational feedback loop (TTFL) is considered the fundamental block of every circadian clock and has been identified in every organism studied so far [9], although some exceptions have been reported very recently [10]–[12].

In mammals, the core clock components are CLOCK, BMAL, PER and CRY. During the day a CLOCK:BMAL heterodimer activates the transcription of the *per* and *cry* genes. Once translated, PER:PER and PER:CRY dimers are formed and then translocate to the nucleus by dusk. These dimers interfere negatively with CLOCK:BMAL and therefore repress the transcription of their own genes. Once the PER:CRY dimer is degraded, CLOCK:BMAL can again induce transcription of *per* and *cry*, thus closing the loop. Posttranscriptional regulation also plays a role fine tuning the circadian molecular machinery. For example, casein kinase 1 epsilon (CKIε) also regulates the clock fulfilling three roles: 1) tagging PER monomers for degradation; 2) promoting PER:PER and PER:CRY translocation to the nucleus; and 3) it is involved in the degradation of this dimers once they have accomplished their repressive roles [13].

In the case of *Drosophila*, the process presents subtle changes regarding the mammalian machinery. The dimer that activates *per* transcription is CLOCK:CYCLE, which also drives TIMELESS (TIM) transcription; TIM:PER dimers accumulate in the cytoplasm, enter the nucleus and repress CLOCK:CYCLE action and, hence *per* and *tim* transcription. The dCRY protein acts as a photoreceptor and, upon light stimulation, blocks the activity of the PER-TIM dimmer by direct interaction with TIM [14].

The homolog to CKIε in flies, DOUBLETIME (DBT), is in charge of tagging PER monomers in the cytoplasm. At dawn, CRY is activated and induces the degradation of TIM in the PER:TIM dimers. At the same time, DBT induces the degradation of PER [15].

In *Neurospora crassa*, the transcription of the *frq* gene is induced by the WHITE COLLAR (WC) complex, composed of the WC1 and WC2 proteins. The FRQ protein interacts with an RNA helicase, FRH, and this complex then represses the transcription of FRQ. The VIVID photoreceptor acts in a similar way to dCRY: it promotes the degradation of dCLOCK, interacting with the negative element FRQ:FRH and the positive element, the WC complex. Even though the clock proteins of *Neurospora* are not conserved in insects and mammals, they share certain domains, such as the Per-Arnt-Sim (PAS) domain found in PER, CLOCK, WC1-2 and VIVID. Regardless of the actors it is interesting to note that the central TTFL oscillator is very similar in these very different organisms [16]. A similar loop can be found in plants, where TOC1 is repressed by the negative elements LHY y CCA1 [17]. In fact, the same clock architecture is found in the cyanobacteria *Synechococcus aureus*, where the KAI-A protein activates the transcription of the *KaiBC operon*, and the KAI-C product represses it [18]. The cyanobacterial clock can, however, remarkably sustain its rhythmicity in the absence of transcription by means of posttranslational mechanisms, in a loop involving the three KAI proteins [9], [19].

The molecular mechanism of the *C. elegans* circadian clock has yet to be elucidated. Previous bioinformatics approaches have identified possible clock homologs. These include the PER homolog protein LIN-42, and the CLOCK homolog protein AHA-1 [20]. Both these proteins have been described to play a key role in the developmental program of *C. elegans* [21]–[23]. Also, a global genome wide transcriptomics analysis of *Caenorhabditis elegans* N2 nematodes entrained to light:dark (LD) and temperature (T:t, T = high temperature and t = low temperature) has recently shown that several genes are expressed in a circadian manner [24]. The datasets describe that 1817 transcripts are Tt driven; 775 are LD driven; 286 are entrained by Tt and; 406 are entrained by LD. Further analysis of the data showed that the “driven” datasets share 107 transcripts and only 2 genes are entrained by both *Zeitgebers* (*wdr-5.3*, a homolog of mWDR5, a member of a histone methyltransferase complex that in mammals associates with mPER1; and *Y102A5C.6*, a pseudogen). One interpretation could be that *C. elegans* actually has 2 different clocks (differentially entrained by light and temperature). Another interesting and surprising result is that in the conditions used in that report, the mRNA of homologs to clock genes from other organisms does not appear to cycle in *C. elegans*, suggesting that a novel circadian molecular mechanism for nematodes.

There are also some candidates for the entrainment mechanism, including a role for TAX-2, a CNG channel involved in the transduction of temperature and light signals. A mutant strain carrying a mutation in *tax-2* (PR671 strain) showed severely affected transcriptional rhythms. This was studied using a GFP reporter that can be entrained to temperature cycles (*nlp36*p::*gfp*) and three randomly chosen light-entrainable transcripts. This showed that *tax-2* is necessary to convey light and temperature signals to the clock. It is interesting to note that *lite-1* mutant nematodes (a gene that encodes for the LITE-1 photoreceptor), required for low wavelength light responses, did not show any problems in light synchronization [24].

The bioinformatics efforts performed to search for homologs of the clock genes of other species in *C. elegans* have not been very exhaustive and were performed by searching for protein alignment using the BLASTP algorithm, with mammalian and insect proteins as queries [20], [25]. Nowadays, other techniques can be applied, based in probabilistic methods, which might yield more powerful results than those from BLASTP protein sequence remote similarity search tools. Since the introduction of the BLAST suite in the 1990s, several theoretical advances in the homology search methodology have been made, such as hidden Markov models (HMMs), which allow for searches with probabilistic inference technology that are as fast as BLAST [26]–[28]. In this way, hidden Markov models can be generated out of protein alignments of similar proteins of known function from different organisms (i.e., the PERIOD proteins of several insects). With this HMM as input a search can be performed in whole proteomes using the *HMMsearch* included in the HMMER suite [28], [29]. Also, several complete genomes and proteomes from different species of nematodes of the genus *Caenorhabditis* (including *C. elegans*) and other genus, such as *Ascaris* and *Brugia* [30], are completely annotated. By combining the set of tools of the HMMER suite and the availability of these complete proteomes, we have been able to perform exhaustive searches of core clock and accessory genes similarity between the known model species and the proteomes of 19 nematode species.

Hidden Markov Models (HMM) generated from protein alignments of the clock genes of the aforementioned model organisms allow us to determine similar proteins in nematodes. These HMM models contain the most relevant information (protein domains) that allows these proteins to function as components of the molecular clock. By means of these methods, proteins that fit each generated HMM model can be found in *Caenorhabditis elegans* and other nematodes. In this way, we can determine components shared with the *phylum Nematoda* that could be part of a TTFL biological clock.

This type of approach conveys high predictive value to perform experiments to validate the role of particular genes in the circadian clock of *C. elegans*.

## Results

### The proteome of *C. elegans* contains proteins with similitude to clock components from known model organism

**Figure 1:**
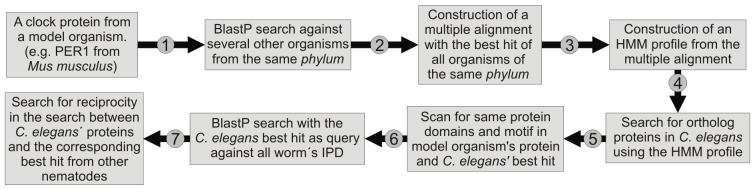
Methodology workflow. The diagram briefly describes the methods we applied in this work.

Using the workflow depicted in figure 1, we first analyzed the existence of clock components similar to those from the most well-known circadian model organisms of 5 different *phyla*: *Arabidopsis thaliana* (plant), *Synechococcus elongatus* (cyanobacteria), *Neurospora crassa* (fungi), *Drosophila melanogaster* (insect), *Mus musculus* (mammal), in *C. elegans* and then the corresponding ortholog proteins in the rest of the nematodes used in this work.

*C. elegans* is very distantly related to plants as well as to cyanobacteria. As expected, nematodes do not exhibit similarity to any of the core clock proteins of either plants or prokaryotes.

It is interesting to note that the search for similar core clock proteins of fungi revealed, quite unexpectedly, several candidate proteins in the proteome of *C. elegans* that presented a significant similarity to each of the components of the fungal clock, as can be seen in table 1.

**Table 1:** Protein homologs to core clock proteins of plants, cyanobacteria and fungi.

Organism	Protein	Clock function	*C. elegans* homolog	E value
*Arabidopsis thaliana*	CCA1	central clock	*None*	*None*
	COP1	LHY/CCA1 modulator	*None*	*None*
	DET1	LHY/CCA1 modulator	*None*	*None*
	GI	central clock	*None*	*None*
	LHY	central clock	*None*	*None*
	LUX	central clock	*None*	*None*
	PRR5	central clock	*None*	*None*
	PRR7	central clock	*None*	*None*
	PRR9	central clock	*None*	*None*
	TOC1	central clock	*None*	*None*
*Synechococcus elongatus*	KAI-A	central clock	*None*	*None*
	KAI-B	central clock	*None*	*None*
	KAI-C	central clock	*None*	*None*
*Neurospora crassa*	FRH	central clock	W08D2.7 (MTR-4)	0
	FRQ	central clock	*None*	*None*
	FWD-1	FRQ modulator	K10B2.1 (LIN-23)	1.2e-80
	VVD	central clock	F16B3.1 (EGL-2)	1.3e-06
	WC-1	central clock	F16B3.1 (EGL-2)	1.1e-05
	WC-2	central clock	F38A6.3d (HIF-1d)	3.9e-10

These proteins are the best hits found by probabilistic inference search based on Hidden Markov Models built upon multiple protein alignments of the core clock proteins of plants (*A. thaliana*), prokaryotes (*S. elongatus*) and fungi (*N. crassa*). Common *C. elegans* names are enclosed between parenthesis when available.

FRH, an RNA helicase that interacts with FRQ, had a practically perfect match (E = 0) with the *C. elegans* protein ceMTR-4. Both proteins have almost the same length (1006aa and 1026aa, respectively) and the same domains, with the exception of a calcium binding domain (Prosite PS00018, calcium binding domain, *EF-Hand 1*). Nevertheless, in *C. elegans* this protein appears to be involved in the polyadenilation of mRNAs destined to degradation or exosomal trimming and is also a substrate of the RTK-RAS-ERK pathway in vivo [31], [32].

FWD-1, a protein involved in FRQ degradation, exhibited a high degree of similarity (E = 1.2×10^−80^) with ceLIN-23 (K10B2.1). These proteins are likely to play a similar role in both organisms since they are both proteins with F-Box and WD40 repeats domains. In *C. elegans*, ceLIN-23 is a component of the SCF ubiquitin ligase complex (Skp1, Cullin, F-Box) which is involved in ubiquitin mediated protein degradation. Also, ceLIN-23 is a negative regulator of postembryonic cell division in all tissue types. It also regulates neurite growth in some types of neurons and the abundance of the glutamate receptor ceGLR-1 [33]–[36].

VIVID (VVD), another negative element of *N. crassa's* circadian clock, present similarity (E = 1.3×10^−6^) with ceEGL-2 (F16B3.1). VVD is a 186 aa protein which contains a PAS domain, that represses light input and regulates the time setting of the circadian clock. On the other hand, ceEGL-2, is a 956aa potassium channel required for egg laying, muscle activation, defecation, mechanosensation and chemosensation [37]–[40].

The *N. crassa* clock has two positive elements that comprise the WC complex, WC1 and WC2. WC1 presents similarity (E = 1.1×10^−5^) to a small region of ceEGL-2, probably due to the fact that they both share a PAS domain. WC2, on the other hand, presents similarity to ceHIF-1d (E = 3.9×10^−10^). In *C. elegans*, this protein is involved in oxygen sensing and is required for nematode survival in hypoxic environments (<1% oxygen) [41]. These two proteins probably do not play the same role in both organisms given the fact that WC2 possesses other domains that are absent in ceHIF-1, such as the GATA zinc finger DNA binding.

### *C. elegans* proteome exhibits similarity to insect and mammalian clock proteins

*C. elegans'* proteome includes many proteins with high similarity to those of the core circadian clock of arthropods. The best hits found with the searches performed using the HMM profiles derived from insect protein alignments [15], [42], [43] are shown in table 2.

**Table 2:** Homolog proteins to core clock genes of insects.

Organism	Protein	Clock function	*C. elegans* homolog	E value
*Drosophila melanogaster*	PER	central clock	F47F6.1b (LIN-42b)	2.10E-30
	TIM	central clock	Y75B8A.22 (TIM-1)	3.20E-18
	CLK	central clock	C25A1.11a (AHA-1a)	1.70E-21
	CYC	central clock	C25A1.11a (AHA-1a)	4.10E-75
	CRY	central clock	*None*	*None*

These proteins are the best hits found by probabilistic inference search based on Hidden Markov Models built upon multiple protein alignments of the core clock proteins of insects (*D. melanogaster*). Common *C. elegans* names are enclosed between parenthesis when available.

The Period (PER) and Timeless (TIM) proteins constitute the principal negative feedback loop of the circadian clock of *Drosophila melanogaster* and other insects. Both proteins have high homology counterparts in *C. elegans'* proteome. ceLIN42b (F47F6.1b) exhibits high similarity to PER (E = 2.1×10^−30^). Both proteins possess PAS domains (PFAM PF00989) and a domain recognized as *PERIOD CIRCADIAN PROTEIN* (PANTHER PTHR11269), although they differ in length: PER has 1218aa and ceLIN-42b is 597aa long. ceTIM-1(Y75B8A.22) presents high similarity to TIM. These proteins are almost the same length (1353aa and 1398aa, respectively) and share the same domains according to analysis by InterProScan. It has been reported, however, that both nematode proteins, ceLIN42 and ceTIM-1, perform heterochronic functions in *C. elegans* [21], [44].

The positive elements, Clock (CLK) and Cycle (CYC), show the best hits with the same protein in *C. elegans*: ceAHA-1A (C25A1.11A), with E = 1.70×10^−21^ and E = 4.1×10^−75^, respectively. This is the only protein that has all the domains present in both CLK and CYC. The analysis by InterProScan reveals 2 PAS domains (SMART SM00091), a bHLH domain (PFAM PF00010), nuclear translocation domains (PROSITE PR00785) and one *CIRCADIAN PROTEIN CLOCK/ARNT/BMAL/PAS* domain (PANTHER PTHR23042), among others. The length of the protein is more similar to that of CYC, which is 413aa long, while CLK is 1023aa long (ceAHA-1A is 453aa long). This protein forms heterodimers with other PAS containing proteins, such as ceAHR-1, ceHIF-1 and ceCKY-1, and is also probably capable of forming homodimers, to then stimulate the transcription of its target genes [45], [46]. This is also the case in flies, where CLK-CYC dimers activate the transcription of the negative factors and other clock controlled genes [15].

Strikingly, *C. elegans* shows no homologs to the Cryptochrome protein (CRY), involved in blue light photoreception and TIM degradation, nor to CRY2, a potent repressor of CLK-CYC mediated transcription, but insensitive to blue light [43]. It is, however, interesting to note that in recent years a novel family of photoreceptors, unknown to other species, has been described in *C. elegans*. This nematodes exhibit a negative phototactic behavior towards UV and blue light. This response is mediated, at least in part, by the LITE-1 photoreceptor, a G protein coupled receptor with similarity to the gustatory receptors of *Drosophila melanogaster*. This protein activates a guanlyate cyclase, producing cGMP, upon activation by low wavelengths of light and, once cGMP levels reach a certain concentration, cGMP-sensitive CNG channels are opened and stimulation of photoreceptor cells occurs. [47].

*C. elegans* also expressed proteins with great similarity to those of the core circadian clock of mammals [48]. The list of the best hits can be found in Table 3.

**Table 3:** Protein homologs to the core clock proteins of mammals.

Organism	Protein		Clock function	*C. elegans* homolog	E value
*Mus musculus*	PER	PER1	central clock	F47F6.1c (LIN-42c)	7.50E-17
		PER2	central clock	F47F6.1b (LIN-42b)	2.90E-21
		PER3	central clock	F47F6.1b (LIN-42b)	5.50E-20
	BMAL	BMAL1	central clock	C25A1.11a (AHA-1a)	1.80E-65
		BMAL2	central clock	C25A1.11a (AHA-1a)	5.40E-63
	CLK		central clock	C25A1.11a (AHA-1a)	1.50E-17
	NPAS2		central clock	C25A1.11a (AHA-1a)	6.80E-19
	CRY	CRY1	central clock	*None*	*none*
		CRY2	central clock	*None*	*none*

These proteins are the best hits found by probabilistic inference search based on Hidden Markov Models built upon multiple protein alignments of the core clock of mammals (*M. musculus*). Common *C. elegans* names are enclosed between parenthesis when available.

The positive elements of the mammalian clock are the proteins Clock (CLK) and BMAL. BMAL is similar to dmCYC and there are two variants, BMAL1 and BMAL2. Besides CLK there is also another protein, NPAS2, capable of heterodimerizing with BMAL, and activating the transcription of target genes. The *C. elegans* protein with greatest similarity to CLK, BMAL1, BMAL2 and NPAS2 is ceAHA-1a, with E values of 1.5×10^−17^, 1.8×10^−65^, 5.4×10^−63^ and 6.8×10^−19^, respectively. These results are similar to those found in the case of the insect clock, where the protein with best similarity to CLK and CYC was also ceAHA-1a.

The negative elements of the mammalian clock are the proteins Period (PER) and Cryptochrome (CRY). In this case, 3 PER proteins have been described in mammals: PER1, PER2 y PER3. Once again, as was the case with the insect PER protein, the similar to PER2 and PER3 is ceLIN-42B, with an E value of 2.9×10^−21^ and 5.5×10^−20^, respectively. PER1, on the other hand, was most similar (E = 7,5×10^−17^) to ceLIN-42C. There are no CRY similarities in *C. elegans*.

Even though the similarity scores are high, those found in the case of the insect core clock were higher (compare tables 2 and 3).

### *C. elegans* also conserves many insect and mammalian clock modulators/regulators

Since proteins with high degree of similarity to arthropod core clock proteins were found in *C. elegans*, we broadened the search to include known circadian accessory proteins of *Drosophila melanogaster* [42], [49].

The proteins that form accessory loops were the first candidates to search for. These proteins are Vrille, Par Domain Protein 1E and Clockwork Orange.

Vrille (VRI) belongs to the 2nd negative feedback loop of the clock that inhibits CLK transcription. A similar protein, ceATF-2 (K08F2.2), with a score of E = 1.7×10^−17^ was found. Domain analysis shows that both proteins possess a bZIP domain (PFAM PF07716). It is known that ceATF-2 is a transcription factor that negatively regulate the autophagy genes ceBEC-1 y ceLGG-1 [50], but it is unknown whether it regulates ceAHA-1 (similar to CLK, see table 2) or not.

Par Domain Protein 1e (PDP1e), is involved in the 2nd positive feedback loop of the fly's clock and fulfills an antagonistic role to that of VRI, promoting CLK expression. In this case, ceCES-2 is the most similar protein, with an E = 2×10^−6^. Both proteins possess bZIP (PFAM PF07716) domains, although their sizes are very different. While PDP1e is 647aa long, ceCES-2 is only 211 residues long. It is known that this protein regulates apoptosis in the nematodes and that it is capable of forming dimers with ceATF-2 [51], [52].

Clockwork Orange (CWO) is part of a third loop and acts as a transcriptional repressor of CLK target genes, thus modulating the amplitude of Drosophila's circadian clock controlled genes' oscillations. No similarities were found in *C. elegans*.

Clock modulating proteins were then studied: CREB, DoubleTime (DBT), Casein kinase 2 (CK2), Shaggy (SGG), the protein phosphatases 1 and 2 (PP1 y PP2), Supernumerary Limbs (SLMB), Rhodopsin (Rh), Nemo (NMO), Nocte and phospholipase C (NORPA). The similarities and function of these proteins are summarized in table 4.

**Table 4:** Homolog proteins to accessory proteins of the insect circadian clock.

Organism	Protein		Clock function	*C. elegans* homolog	E value
*Drosophila melanogaster*	VRI		2nd loop	K08F8.2 (ATF-2)	1.70E-17
	PDP1E		2nd loop	ZK909.4 (CES-2)	2.00E-06
	CWO		3rd loop	*None*	*none*
	CREB		Modulator	Y41C4A.4d (CRH-1d)	8.10E-43
	DBT		Modulator	C03C10.1 (KIN-19)	5.30E-190
	CK2A		Modulator	B0205.7 (KIN-3)	3.50E-209
	CK2B		Modulator	T01G9.6b (KIN-10)	1.50E-121
	SGG		Modulator	Y18D10A.5 (GSK-3)	3.30E-189
	PP1	PP1b	Modulator	F29F11.6a (GSP-1a)	1.30E-216
		PP1-13C	Modulator	F56C9.1 (GSP-2)	1.30E-218
	PP2	PP2a_MTS	Modulator	Y75B8A.30 (PPH-4.1)	3.90E-177
		PP2a_TWS	Modulator	F26E4.1 (SUR-6)	4.00E-213
		PP2a_WBT_A	Modulator	W08G11.4 (PPTR-1)	4.30E-256
		PP2a_WBT_B	Modulator	C13G3.3c (PPTR-2c)	9.50E-279
	PRMT-5		Modulator	C34E10.5 (PRMT-5)	3.70E-144
	SLMB		Modulator	K10B2.1 (LIN-23)	1.90E-232
	RH1		Modulator	**C52B11.3 (DOP-4)**	**8.60E-34**
	RH5		Modulator	**C52B11.3 (DOP-4)**	**4.00E-30**
	RH6		Modulator	**C52B11.3 (DOP-4)**	**8.40E-30**
	NMO		Modulator	W06F12.1e (LIT-1e)	1.50E-182
	NOCTE		Modulator	*None*	*None*
	NORPA		Modulator	B0348.4a (EGL8a)	0
	TO		Output	*None*	*None*
	PDF		Output	T07E3.6b (PDF-1)	3.00E-02
	PDFR		Output	C13B9.4a (PDFR-1a)	1.80E-127
	SLO		Output	Y51A2D.19a (SLO-1a)	0
	NA		Output	C11D2.6a (UNC-77a)	0
	IR		Output	M02A10.2a (IRK-2a)	8.50E-159
	JET		Output	C02F5.7a	8.80E-128
	CSN4		Output	Y55F3AM.15 (CSN-4)	7.80E-85
	PKA		Output	ZK909.2h (KIN-1h)	1.60E-217
	EBONY		Output	**Y66D12A.14**	**1.40E-60**
	LRK		Output	F18H3.3b (PAB-2b)	1.80E-36
	FER2		Output	B0304.1b (HLH-1b)	1.90E-12
	JAFRAC		Output	F09E5.15a (PRDX-2a)	5.10E-105
	WDS-PB		Output	C14B1.4 (WDR-5.1)	3.50E-165

These proteins are the best hits found by probabilistic inference search based on Hidden Markov Models built upon multiple protein alignments of the accessory proteins of the circadian clock of insects (*D. melanogaster*). Those proteins that did not possess the same domains that the query profile are highlighted (bold). Common *C. elegans* names are enclosed between parenthesis when available.

Proteins related to insect clock output were then studied. These include Takeout (TO), Pigment Dispersing Factor (PDF), Slowpoke (SLO), Narrow Abdomen (NA), Inward Rectifier (IR), Jetlag (Jet), COP9 signalosome (CSN4), Protein kinase A (PKA), Beta Alanil Conjugase Synthase (EBONY), Lark (LRK), Transcription factor FER2 (FER2). The similarities and functions of these proteins are summarized in table 4.

Even though the homology score was higher in the case of the insect core clock proteins, *C. elegans'* proteome was also searched for the mammalian accessory proteins [48].

The positive elements of the second loop are ROR-A and ROR-B. They act positively on BMAL transcription. The protein with higher similarity to ROR-A is ceNHR-23b (C01H6.5b), with E = 1.4×10^−70^; and the similar to ROR-B is ceNHR-23a (C01H6.5a), with E = 4.5×10^−73^. In the nematodes this protein acts as a nuclear hormone receptor required for larval molting [53].

REB-ERV alpha (NR1D1 and NR1D2) acts as the negative element of the second loop, inhibiting BMAL transcription. The closest protein similar to NR1D1 and NR1D2 is ceNHR-85a (W05B5.3a), with E values of 8.1×10^−47^ and 8.7×10^−55^, respectively. This protein is a nuclear hormone receptor involved in egg laying and SDS resistant dauer larva formation [54].

DBP (D-Box binding protein) is part of a second accessory loop, acting through binding to D-Box elements of target genes [55]. The similar to DBP is ceCES-2 (ZK909.4), with E = 1.5×10^−18^, which is also the homolog to dmPDP-1e.

We also studied clock-modulating proteins such as CASEIN KINASE 1A, CASEIN KINASE 1D, CASEIN KINASE 1E, DEC1, DEC2, FBXL3, NOCTURNIN (CCRN4L), PPARGC1A. The similarities and functions of these proteins are summarized in table 5.

**Table 5:** Homolog protein to accessory proteins of the mammalian circadian clock.

Organism	Protein		Clock function	*C. elegans* homolog	E value
	ROR	ROR-A	2nd loop	C01H6.5b (NHR-23b)	1.40E-70
					
*Mus musculus*	REB-ERVa	ROR-B	2nd loop	C01H6.5a (NHR-23a)	4.50E-73
		NR1D1	2nd loop	W05B5.3a (NHR-85a)	8.10E-47
		NR1D2	2nd loop	W05B5.3a (NHR-85a)	8.70E-55
	DBP		3rd loop	ZK909.4 (CES-2)	1.50E-18
	CSNK	CSNK1a	Modulator	C03C10.1 (KIN-19)	4.30E-204
		CSNK1d	Modulator	F46F2.2b (KIN-20b)	1.40E-172
		CSNK1e	Modulator	F46F2.2b (KIN-20b)	5.00E-172
	CCRN4L		Modulator	ZC518.3c (CCR-4c)	3.50E-21
	DEC1		Modulator	**Y54G2A.1 (LIN-22)**	**4.20E-05**
	DEC2		Modulator	**Y54G2A.1 (LIN-22)**	**1.50E-05**
	FBXL-3		Modulator	C02F5.7a	5.20E-04
	PPARGC1a		Modulator	**F18H3.3b (PAB-2b)**	**6.10E-08**
	MEL receptor	MEL1A	Output	F41E7.3 (NPR-6)	1.00E-26
		MEL1B	Output	F41E7.3 (NPR-6)	1.10E-31
	OPN4		Input	**T11B7.4e (ALP-1e)**	**5.20E-61**
	PKC2		Output	*None*	*None*
	VIP		Output	*None*	*None*
	VPAC2		Output	C18B12.2 (SEB-3)	1.60E-37
	PRDX-2		Output	F09E5.15a (PRDX-2a)	1.70E-104
	WD-REP		Output	C14B1.4 (WDR-5.1)	2.70E-159

These proteins are the best hits found by probabilistic inference search based on Hidden Markov Models built upon multiple protein alignments of the accessory proteins of the circadian clock of mammals (*M. musculus*). Those proteins that did not possess the same domains that the query profile are highlighted (bold). Common *C. elegans* names are enclosed between parenthesis when available.

We then looked for the proteins related to input and output of the clock, including melatonin receptors MEL1A and MEL1B, MELANOPSIN (OPN4), PROKINETICIN (PKC2), VIP and VIP receptor (VPAC2). The similarities and function of these proteins are summarized in table 5. It is interesting to note that previous work from our lab described the existence of melatonin synthesis rhythms and in the ASMT enzyme, which is the rate limiting enzyme of the process [56]. However, the receptors for this humoral signal remain undiscovered. Here we report several melatonin receptor candidates; the one with the highest degree of similarity to the mammalian receptor is ceF41E7.3. This protein is an orphan neuropeptide receptor and could certainly be tested experimentally to verify whether it plays a role in melatonin signaling.

### *C. elegans'* proteome exhibits high similarity to clock accessory proteins of insects and mammals

The previous results show that *C. elegans* expresses the proteins needed to build a clock similar to that of insects with the important exception of a protein similar to dmCRY, the protein responsible for TIM degradation upon blue light stimulation [42]. In the mammalian case, mCRY is part of the core of the clock itself [13].

In the case of clock accessory proteins, *C. elegans* exhibits similarities to form a complete secondary insect-like loop and also most of the insect modulatory and output proteins, with the exception of rhodopsins, dmNOCTE, dmTO y dmEBONY. However, no similarities to dmCWO were found, a protein involved in the third loop of the insect clock. We also found similarities to the proteins of the secondary loops and several of the accessory proteins of the mammalian clock. It is interesting to point out that there were no similarities to VIP, a neuropeptide involved in the synchronization of the mammalian clock neurons, although our methods indicated similarities to its receptor VPAC2. In insects, functions analog to VIP neuropeptide appear to be encoded by PDF. *C. elegans* present two PDF similarities and also a protein similar to a PDF receptor [57], [58]. It is tempting to consider PDF as a putative output protein for the C. elegans circadian clock, a possibility that remains to be determined experimentally.

The insect and mammalian similarity tables (see tables 2–5) include several accessory proteins with highly elevated and significant E values. This can be explained by the fact than many of those proteins are ionic channels, phosphatases or kinases, all of which are very conserved throughout evolution [59]–[66] and hence high E values were expected. On the other hand, proteins that are more clock-specific, as is the case of PRMT-5 (*PROTEIN ARGININE METHYL TRANSFERASE 5*), bear more interest in this type of predictive studies. This protein, which is highly similar (E = 3.70E-144) to the *C. elegans* protein C34E10.5, modulates the alternative splicing of clock genes in *D. melanogaster and A. thaliana*. Null mutants of this enzyme present severely affected rhythms in both species [67], [68]. It would be interesting to study how this protein affects the circadian behavior of *C. elegans*.

### Common clock components in the *phylum Nematoda* and phylogenetic studies

Once that it was established that the proteome of *C. elegans* contained proteins with similarity to insect and mammalian clock proteins, we searched for homolog proteins in the complete proteomes of other nematodes: *Ascaris suum*, *Brugia malayi, Bursaphelenchus xylophilus, Caenorhabditis angaria, Caenorhabditis brenneri, Caenorhabditis briggsae, Caenorhabditis japonica, Caenorhabditis remanei, Caenorhabditis sp7, Caenorhabditis sp9, Caenorhabditis sp11* and *Pristionchus pacificus*. The results are summarized in Tables S1 and S2. Thirteen mammalian and thirty two insect *C. elegans'* protein hits were also found in the other nematode proteomes that were analyzed. Interestingly there were 7 proteins common to mammals, insects and nematodes. These common proteins include two core clock components, the positive core clock element Cycle/BMAL and the negative element Period; and four modulators, the circadian clock kinase Dbt/CSNK4; the F-Box modulator Jetlag/FBXL; the transcriptional coactivator Lark/PPARGC1-α; the peroxirredoxin Jafrac/PRDX-2 and the WDR5 histone modification proteins Wds/WD-Rep. The best conserved protein hits can be seen in table 6 and figure S2.

**Table 6:** Common elements of the insect and mammalian clock also found in nematodes.

Organism	Protein						
***M. musculus***	BMAL1	CSNK1a	FBXL	PPARGC1a	PER	WD-Rep	PRDX-2
***D. melanogaster***	Cyc	Dbt	Jetlag	Lark	Period	Wds-PB	Jafrac
***C. elegans***	C25A1.11a	C03C10.1	C02F5.7a	F18H3.3b	F47F6.1c	C14B1.4	F09E5.15a
*A. suum*	GS_18509	GS_19252	GS_19076	GS_22069	GS_08634	GS_18544	GS_23521
*B. malayi*	BM21318	BM20483	BM06465	BM03094	BM06378	BM03793	BM19146
*B. xylophilus*	BUX.s00397.112	BUX.s00055.190	BUX.s00422.570	BUX.s00579.708	BUX.s00397.112	BUX.s00579.553	BUX.s01109.415
*C. angaria*	CAN07933	CAN15839	CAN15035	CAN13338	CAN12189	CAN22866	CAN05705
*C. brenneri*	CBN21788	CBN06321	CBN03873	CBN18184	CBN20940	CBN24532	CBN11882
*C. briggsae*	CBG12219	CBG20206	CBG16659	CBG07431	CBG07211	CBG09206	CBG25150
*C. japonica*	CJA09659	CJA17198	CJA04910	CJA16600	CJA16851	CJA10837	CJA14459
*C. remanei*	CRE23963	CRE13355	CRE25417	CRE22405	CRE17943	CRE25440	CRE11768
*C. sp7*	g19865	g29573	g22800	g33812	g31054	g26350	g24613
*C. sp9*	g12945	g44344	g29591	g23257	g23334	g4010	g38213
*C. sp11*	g9507	g14251	g435	g1414	g4740	g7816	g9373
*P. pacificus*	PPA09278	PPA25500	PPA20677	PPA20102	PPA04587	PPA00309	PPA28346

These proteins are the best hits found by probabilistic inference search based on Hidden Markov Models built upon multiple protein alignments of the accessory proteins of the circadian clock of mammals (*M. musculus*) and insects (*D. melanogaster*). Those proteins that did not possess the same domains that the query profile are highlighted in red.

**Figure 2:**
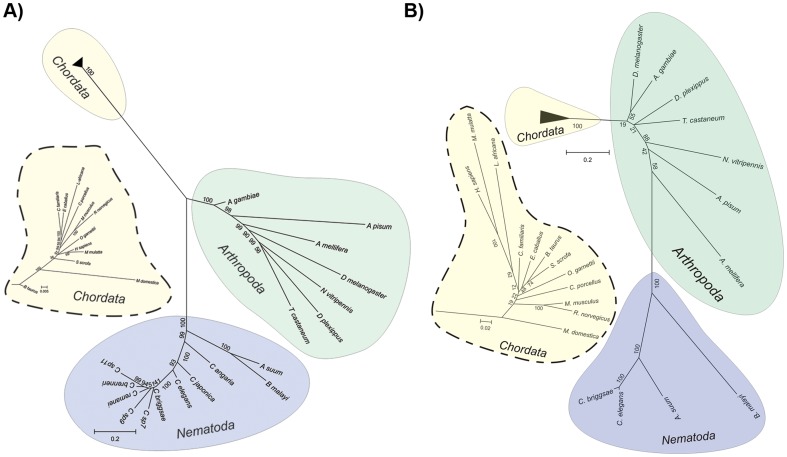
Phylogenetic analysis. A) Phylogenetic tree of the conserved clock proteins of insects, mammals and nematodes. B) Phylogenetic tree based on Cytochrome B protein sequences of insects, mammals and nematodes. The insets show a zoomed in view of the Chordata phylum branches.

We also studied the evolutionary relationships between these clock proteins conserved among the three different phyla. Concatemers of these 7 proteins for different species of the three taxa were built from the respective protein alignments and a phylogenetic tree was constructed as described in the materials and methods section. A cytochrome B phylogenetic tree was also built for comparison purposes. As can be seen in figure 2 both trees show three clearly distinct clades, one belonging to each phylum.

There is a longstanding debate about *C. elegans'* relationship with other model organisms [69]. There are two hypotheses, the *coelomata* hypothesis indicates that nematode are closer to insects than to mammals; and the *ecdysozoa* hypothesis states that insects and mammals are closer to each other than either of them to *C. elegans*; indeed, there is evidence supporting both hypotheses. Studies based on 18S RNA sequence state that the nematodes are more closely related to animals that go through the process of ecdysis. This hypothesis, however, depends on the exclusion of all nematodes with the exception of *Trichinella spiralis*. If the rest of the nematodes are included, then they group in a clade consistent with the *coelomata* hypothesis [70]. RNApol 2 sequence-based trees also support this hypothesis [71]. As shown in figure 2, our results also favor this hypothesis, suggesting that *D. melanogaster* and *M. musculus* are closer to each other, than either of them to *C. elegans*.

**Figure 3:**
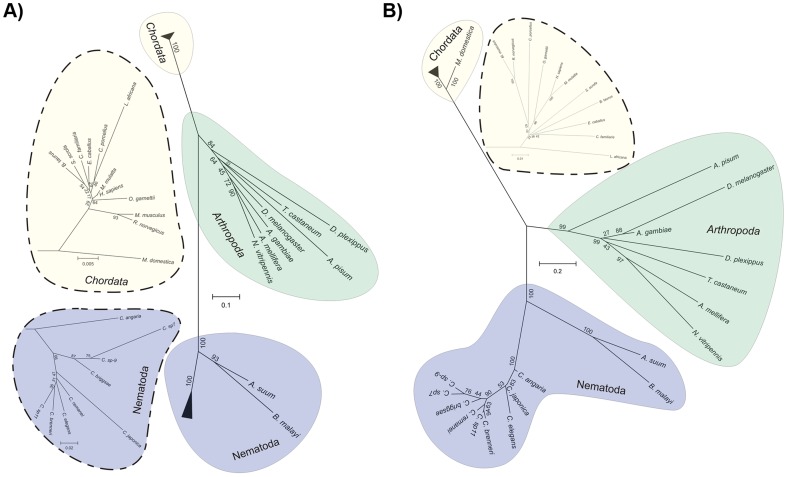
Core clock proteins phylogenetic analysis. A) Phylogenetic tree of the conserved proteins mmBMAL, dmCYCLE and its homolog ceAHA-1. B) Phylogenetic tree of the conserved PERIOD proteins. A zoomed in view of the branches corresponding to the Chordata (A and B) and Nematoda (A) phylum are shown in the insets.

The analysis of trees based on a single core clock protein indicates the same kind of grouping (figure 3). One interesting case is that of the TIMELESS homolog. ceTIM-1 is closer to dmTIMEOUT and to mmTIMELESS, that to dmTIMELESS (figure S1). In mammals, this protein is essential for embryonic development and is associated to DNA metabolism. It is also known to associate with peroxirredoxin 2 during cell cycle check points [72]. In *Drosophila*, TIMEOUT is also involved in DNA metabolism, chromosomal cohesion and circadian photoreception [73]–[75]. This means that *C. elegans'* similarity to TIMELESS is closer to the proteins that perform developmental roles rather than to the protein involved in circadian rhythms; indeed, there is experimental evidence to support ceTIM-1's role in chromosomal cohesion and developmental timing [44], [76], [77].

### Circadian *Cis* acting promoter elements analysis

In parallel to the proteins (*trans* acting factors) of the clock, c*is* acting promoter elements modulate the phase of the rhythms found at the mRNA level in other model organisms [78]–[80]. We searched for the most well-known *cis* elements, including the canonic E-Box element, in the promoters of all the genes that codify for proteins similar to insect/mammalian proteins (group 1), insect proteins (group 2) and mammalian proteins (group 3). The analysis revealed several circadian related *cis* acting promoter elements can be found in *C. elegans*.

The morning E-Box element was found to be represented in the promoters of all three groups. This element is bound by bHLH domain containing proteins, such as Cycle/Bmal, and was the first promoter element associated with circadian transcription [55], [81]. This element is found in the promoters of the cycle/bmal, lark/pparg1-α, wds/wd-r-2 and dbt/csnk4 group 1 gene homologs. Strikingly it is not found in the promoter of the period homolog lin-42, although a non-canonic E-Box element is present (data not shown).

The daytime canonic D-Box element was also found in the promoter elements of some of the *C. elegans'* gene homologs (groups 1 and 2). This element is bound by DBP in a repressor-antiphasic-to-activator mechanism [55]. It was found in the promoter of the cycle/bmal of the group 1 genes homologs.

The nighttime canonic RRE element is bound by RevErbA/ROR binding elements in a repressor-precedes-activator pattern (as is the case for the E-Box element) [55], [80]. Interestingly, this element was found in the promoters of the *nhr-85*, the RevErbA homolog, and in the peroxirredoxin-2 gene homolog.

The complete set of results, which includes other circadian expression associated promoter elements (GRE, PPRE, CREB, HSE and CEBP) can be found in Table S3.

## Discussion

The bioinformatics analysis approach used in this *C. elegans* work based on homolog sequence searches from the model organisms *Mus musculus, Drosophila melanogaster, Neurospora crassa, Arabidopsis thaliana* and *Synechoccocus elongatus* yielded some novel and unexpected results that differ from simple BlastP search strategies (Table S4). On the one hand, *C. elegans* expresses similar proteins to the ones from the core clock of three of the studied model organisms: *M. musculus, D. melanogaster* and *N. crassa*. The high homology to mammal and insect core clock proteins is not surprising if we take into account previous reports and results from our own lab. In fact, most of the studies performed on recent years were focused on the role of these components in the *C. elegans* clock. However, the existence of high similarity to the clock proteins of the fungi *N. crassa* is much more unexpected and has not been reported so far to our knowledge. In particular, the perfect similitude between ncFRH and its possible *C. elegans* counterpart, ceMTR-4, is remarkable. These proteins not only share sequence similitude but also exhibit a similar size (1106aa and 1026aa, respectively) and present almost the same protein domains with just one exception (calcium binding domain, EF-Hand 1). It is also notable that ncFWD-1, which is involved in ncFRQ degradation, exhibited strong sequence similitude to ceLIN-23, which has very similar molecular functions already described in the nematode. Nevertheless, the lack of a protein similar to ncFRQ would hinder the function of a *C. elegans* clock based on the fungal TTFL model. Regarding the rest of the central clock components of *N. crassa*, we must also point out that the WC complex proteins (ncWC1 and ncWC2), that act as positive elements, do not appear to have proteins with strong similitude in *C. elegans*. The same is true in the case of ncVIVID. These results suggest that only part of an ancestral clock is conserved in nematodes and some of the elements could have been substituted by some new, yet unknown, elements, thus closing the regulatory loop. Even though the similitude is not perfect and the functions described in *C. elegans* for these proteins is different to those expected, they could also be part of the nematode clock by performing additional functions that have not been discovered yet. Even though there are no experimental records to support this hypothesis, the possibility of similitude between *C. elegans'* clock components and those of *N. crassa's* clock has not been examined. As a first approximation, none of the *C. elegans'* proteins similar to *N. crassa'*s core clock proteins appear to cycle at the mRNA level in the different conditions studied according to the published microarray study [24].

Besides the high similitude to the *N. crassa's* clock, the results obtained after comparing the proteins that make up the insect and mammalian clock were more conclusive. *C. elegans* exhibits not only the required proteins to build the core clock but also some accessory proteins required for circadian rhythms in both the insect and the mammalian molecular clock. Among the sets of core proteins, ceLIN-42b and ceLIN-42c were of great similarity to PER and ceTIM-1 could be the equivalent of the insect TIM clock protein. Also, ceAHA-1a is the sole protein with similarity to the positive elements of the clock (dmCLK/CYC y mmCLK/BMAL) and includes all of the required domains for the circadian functions of these proteins. It must be pointed out, however, that these homolog proteins exert heterochronic and chromosomal cohesion roles in the nematodes [20], [21], [44] and their function in the circadian clock of *C. elegans* is not completely clear.

*lin-42* encodes three different isoforms, LIN-42a, LIN-42b and LIN-42c. The b and c isoforms contain PAS domains such as those found in insect and mammalian PER. The experimental evidence suggests that *lin-42* is involved in larval development controlling the multiple changes that occur in different tissues (hypodermis, gonads, sex myoblasts and vulva) [21], [22], in molting synchronization [82] and in circadian locomotor activity period determination [3]. It is also known that *lin-42* gene expression cycles throughout development [21], [83] and that LIN-42a in particular, whose mRNA peaks during the four larval moltings with a close to 8-h period [82], is required to generate quick, rhythmic and productive molts. In line with this, the forced and constitutive expression of *lin-42a* in wild type nematodes generates anachronistic larval molts and lethargy [82]. The molting defects observed in the *lin-42(ok2385)* (which bears a deletion affecting isoforms a and b) and *lin-42(n1089)* (which bears a deletion affecting isoforms b and c) mutants are completely rescued by *lin-42a* [82]. In addition, the defects in the temporal program of cellular division observed in nematodes carrying the *n1089*, *mg152* and *ve11* mutant alleles can also be fully reversed by the downstream portion of the *lin-42* locus, that codifies for the LIN-42a isoform [22]. However, it must be noted that the three *lin-42* isoforms may interact and influence the pace of larval development. In this sense, the overexpression of LIN-42c causes a dramatically reduction in the pace of development in *lin-42(ok2385)* larvae, suggesting an antagonic role to that of LIN-42a. Also, the a and b isoforms share SYQ and LT domains, potentially performing redundant roles [82]. Taken together, these results suggest that LIN-42a would be the isoform mainly involved in heterochronic functions, leading to speculate that the PAS-containing b and c isoform may fulfill other functions, one of which could be the regulation of circadian rhythms in the nematodes. In accordance to this hypothesis, adult nematodes that lack PAS containing LIN-42 isoforms exhibit a longer locomotor activity circadian period than wild type [3].

ceTIM-1, another possible negative element found in *C. elegans*, has been implied to be only involved in heterochronic functions. RNAi-mediated studies to knock down ceTIM-1 and ceKIN-20 (homolog to dmDBT and mmCSNK4) indicated that both elements are involved in the seam cell terminal differentiation program during the L4 stage [76]. Also, ceTIM-1 is essential for chromosomal cohesion regulation and is involved in chromosomal location determination of the non-SMZ cohesion subunits [44]. It is interesting to point out that in insects a paralog gene to *timeless, timeout*, has been described. *timeless* is a canonic gene of the circadian clock of *D. melanogaster* and other insects, while *timeout* is a multiple-function gene and plays a key role in the maintenance of chromosome integrity, light entrainment of the circadian clock, embryonic development and DNA replication regulation. Studies have shown that in many insects *timeout* circadian expression is light-dependent and also particularly necessary for circadian photoreception in fruit flies [73], [84]. In mammals, there is only one *tim* gene, called *timeless*, that is more similar in sequence to insect *timeout* rather than to insect *timeless* (figure S1). This gene is involved in replication termination and cell cycle progression [85]–[87] and has recently been shown to participate in period determination and circadian rhythmicity [88], [89]. It is also known that its expression is rhythmic in the retina [90]. There is also evidence that hymenopteran insects, including ants, bees and wasps, do not have a *timeless* gene and still have a functional circadian clock [84], [91]. In *C*. elegans, as is also the case in mammals, there is only one *tim* gene (ceTIM-1) that is closer in sequence to *D. melanogaster timeout* (figure S1). It could be speculated that ceTIM-1 might also fulfill multiple roles in *C. elegans* as in the mentioned examples, even though the function of this protein in the inner workings of the circadian clock has yet to be found.

On the other hand, the absence in *C. elegans* of proteins similar to the Cryptochromes (CRY) of both insects and mammals is striking. Nevertheless, and in accordance to the absence of a CRY protein, *C. elegans* also completely lacks photolyases, enzymes involved in DNA repair from which cryptochromes evolved [43], [92]. As mentioned earlier, the photoreceptor function of CRY in *D. melanogaster* could have been replaced by the convergent evolution of the novel photoreceptor family discovered in *C. elegans*, composed by LITE-1 and GUR-3 [47], [93], [94]. Even though there is no direct evidence of the role of these proteins in the photic synchronization of the clock, the CNG channel protein TAX-2, which acts downstream of these photoreceptors, is required for the rhythmic expression of diverse transcripts entrained under light: dark 12 h: 12 h cycles [24]. The absence of CRY in the genome of *C*. elegans could mean that the negative transcriptional loop elements conformed by PER/CRY in mammals could have been replaced in the nematode by a PER/TIM dimer, or LIN-42/TIM-1. On the other hand, given the fact that there is no evidence of interaction between these two proteins, the existence of a novel negative element yet to be described cannot be ruled out. In this sense it is worth noting that even though in most insect clocks the PER/TIM dimer fulfills the repressor role, in other insects it is a PER/CRY dimer that performs the same role, even if they also have a TIM protein [43], which infers a great plasticity in the molecular mechanism of the circadian clock.

The most similar protein to the positive elements of the insect and mammalian clocks is ceAHA-1. This protein belongs to the bHLH/PAS protein family, which have the property of binding as an homo or heterodimer to DNA consensus sequences known as an E-Box. There is experimental evidence showing that ceAHA-1 is capable of physical interaction with at least three proteins by means of their PAS domains (ceCKY-1, ceHIF-1 and ceAHR-1) forming heterodimers that translocate to the nucleus and act as transcription factors. The ceAHA-1/ceAHR-1 dimer binds the TTGCGTG sequence and regulates a subset of genes involved in neuronal development [23], [95]; the ceAHA-1/ceCKY-1 dimer regulates gene expression in the pharynx by binding to the TGCGTG sequence [95], [96]; finally, the ceAHA-1/ceHIF-1 dimer binds to the sequence CACGTA, to modulate the transcription of genes involved in iron homeostasis [97] and to the TACGTG sequence, to modulate the response to hypoxia [41], [95]. There is currently no evidence that links ceAHA-1 to the circadian clock of *C. elegans*; however, given the fact that this protein has bHLH DNA binding domains and PAS protein interaction domains, it would not be surprising if ceAHA-1, acting as a homodimer or as a heterodimer in association with another protein, could play the role of the mammalian CLK/BMAL or insect CLK/CYC heterodimers. Several ceAHA-1-containing dimers regulating the transcription of genes involved in different functions in the nematode have been reported; moreover, the bioinformatics analysis found ceAHA-1a as the most similar protein to the known positive elements. However, there is no experimental evidence that corroborates the existence of ceAHA-1 homodimers in *C. elegans*. In this regard, more experiments will be necessary to study the possible role of ceAHA-1 in the molecular basis of the *C. elegans'* circadian clock.

Recently, a global transcriptomic study of nematodes kept under light:dark and temperature (warm:cold) 24 hour cycles did not find significant cycles in the mRNA of these possible core clock genes homologs [24]. It is important to know that this study was performed using microarrays and hence it is not possible to discriminate between the different isoforms of *lin-42* and *aha-1*, among others. Also, the study was performed analyzing the gene expression levels of whole nematode populations in different timepoints, which could have generated a high level of noise and hence, loss of detection sensibility and the masking of positive results due to the contribution of non-rhythmic tissues. This could have negatively affected the detection of core clock homologs as it is known that in all eukaryotes studied so far, the expression of the core clock genes is rhythmic in certain cells and tissues (and may even change their respective phases in different tissues) and suffer quick dampening in the absence of environmental cues. One such example is the *period* gene of *D. melanogaster*. *period* rhythms were initially described in the heads of adult flies and even though the mRNA in the rest of the fly tissues also shows circadian fluctuations in *per* (with similar phase and amplitude), rhythms from whole body RNA extractions are much noisier. In particular, the *per* rhythm is absent in ovaries, which represents around 70% of the total RNA of female flies. The non-rhythmic *per* mRNA of the ovary represents 26% of the total *per* mRNA and generates low overall *per* rhythms in studies performed with whole female or whole mixed sex flies. Also, *per* rhythms are far more robust in the head under constant conditions than in the rest of the body, and it is a fact that the amplitude of *per* oscillations decreases well over 70% in peripheral clocks after 3 days in constant conditions, whereas they only diminish 30% in head neurons [98], [99]. This observation could be valid for some of the genes analyzed in Van der Linden et al [24]. For example, *aha-1* and *atf-2* were scored as cycling under LD conditions (pF24<0.02) but were not robust enough in constant conditions. However, it is also possible that the possible homologs to core clock genes do not have a circadian expression and *C. elegans* may have a novel circadian clock.

Every approach pursued so far to try to discover the molecular basis that dictates the workings of the *C. elegans* clock were based on the widely studied and well understood classic models of *Drosophila melanogaster* and *Mus musculus*. But even if all clocks described to date are based in a transcriptional translational feedback loop (TTFL), with the exception of the aforementioned redox driven oscillators, the proteins that make up the mechanism of these clocks are not conserved in all the studied model organisms. One example is the filamentous fungi *Neurospora crassa* that has served for nearly half a century as a durable model organism for uncovering the basic circadian physiology and molecular biology. In the molecular clock of this organism, the proteins that serve as the core clock of the TTFL, WC, FRQ, FRH and VIVIV, are not conserved in insects or mammals. The molecular clock of *Neurospora crassa* took years of exhaustive research to be discovered. This was done through genetic screens of mutant phenotypes and homology based sequence searches that allowed, for example, the identification of shared PAS domains between WC1/2 and PER/CLOCK.

On the other hand, the lack of robust circadian rhythms in the expression of the principal candidate homolog genes does not mean that the gene products could not be rhythmic through posttranscriptional regulation mechanisms. In this sense, there are cases of circadian rhythms that respond to the PTFL (post-translational feedback loop) model, that are sustained even in the absence of transcription and translation. One such example is seen in the cyanobacteria *Synechococcus elongatus*, where one of the clock components goes through rhythmic cycles of phosphorylation [100]. Another example of a PTFL mechanism that has gained momentum is based on the superoxidation and reduction of peroxirredoxin (PRX), a family of proteins involved in preventing damage from reactive oxygen species [101]. PRXs are highly conserved in a great number of organisms and though this mechanism was first discovered in mammalian red blood cells and in the unicellular green alga *Ostreococcus tauri* [10], [11], there is evidence that the rhythm in the oxidative state of these proteins is conserved in all the model organisms from cyanobacteria to plants, fungi, fruit flies and mice. Hence, PRXs have been proposed as universal markers for circadian rhythms [12]. Superoxidation of PRXs is dependent on the redox state of the cell and was shown to be rhythmic even in the complete absence of transcription and of some clock genes necessary to TTFL function. The fact that oxidation rhythms of PRXs have been found in organisms that possess functional TTFLs means that these mechanisms are not mutually exclusive, and to date it is not clear whether this system influences or regulates known TTFL-based clocks. Moreover, it is also unclear whether the oscillation in the oxidative state of the peroxirredoxin is itself part of the PTFL-based clock, or rather an output of another oscillator yet to be found. Recently, an oxidation rhythm of peroxirredoxin-2 (PRDX-2) has been found in *C. elegans* kept under constant darkness and temperature. However, *prdx-2* mRNA is not rhythmic. This oxidation rhythm represents a phylogenetically conserved molecular marker of the circadian clock in *C. elegans* [102] and could be part of a still uncharacterized PTFL-based clock. This does not exclude the possibility of *C. elegans* having a classic TTFL clock and the bioinformatics evidence presented in this work supports the existence of such clock and also of its phylogenetic similarity to the mammalian and insect clock.

Many studies have experimentally shown the existence of circadian rhythms in *C. elegans* at the molecular, physiological and behavioral level. Taken together, the data is indicative of the existence of a biological clock in *C. elegans* that responds to environmental signals (light and temperature cues), but so far the efforts have lacked an integrative analysis to allow for the establishment of the mechanism behind the observations, and the formulation of a model that explains how the circadian clock of *C. elegans* works. This could be, in part, due to our own human nature and our strictly scientific way of classifying and contrasting new findings with previous theories. We must take careful consideration in *C. elegans* unique characteristics, such as its lack of eyes, soil-dwelling life, low or no contact with sunlight, which make it very different from all the other model organisms studied so far. Indeed, previous studies and the current bioinformatics analysis should be complemented with experimental data from more robust phenotype screenings or the use of high sensitivity reporters that allow for real time and *in vivo* recording of all the candidate genes found.

## Materials and Methods

### Protein databases

In this work we used the genomes and proteomes deposited in WormBase WS230 (table 7). These include *Caenorhabditis elegans* and 8 other members of the *Caenorhabditis* genus, and 2 other species of the genera *Ascaris* and *Brugia*. The proteomes of all these nematodes were extracted from their corresponding WormBase files (http://www.wormbase.org/pub). We then created an individual proteome database (IPD) with 11 individual proteomic databases containing the annotated proteome of each nematode. All of them were indexed using the formatdb routine from the NCBI Blast standalone suite v2.2.28.

**Table 7:** Organisms used in this work.

**Nematoda**	**Tax. ID**	**Chordata**	**Tax. ID**	**Arthropoda**	**Tax. ID**
***Caenorhabditis elegans***	6239	***Mus musculus***	10090	***Drosophila melanogaster***	7227
*Ascaris suum*	6253	*Bos Taurus*	9913	*Acyrthosiphon pisum*	7029
*Brugia malayi*	6279	*Canis lupus familiaris*	9615	*Anopheles gambiae*	7165
*Bursaphelenchus xylophilus*	6326	*Cavia porcellus*	10141	*Apis mellifera*	7460
*Caenorhabditis angaria*	860376	*Equus caballus*	9796	*Danaus plexippus*	13037
*Caenorhabditis brenneri*	135651	*Homo sapiens*	9606	*Nasonia vitripennis*	7425
*Caenorhabditis briggsae*	6238	*Loxodonta africana*	9785	*Tribolium castaneum*	7070
*Caenorhabditis japonica*	281687	*Macaca mulatta*	9544		
*Caenorhabditis remanei*	31234	*Monodelphis domestica*	13616		
*Caenorhabditis sp11.*	886184	*Ontolemur garnettii*	30611		
*Caenorhabditis sp7.*	870436	*Rattus norvegicus*	10116		
*Caenorhabditis sp9.*	870437	*Sus scrofa*	9823		
*Pristionchus pacificus*	54126				
**Cyanobacteria**	**Tax. ID**	**Ascomycota**	**Tax. ID**	**Tracheophyta**	**Tax. ID**
***Synechococcus elongatus***	32046	***Neurospora crassa***	5141	***Arabidopsis thaliana***	3702
*Acaryochloris marina*	155978	*Ajellomyces dermatitidis*	5039	*Brachypodium distachyon*	15368
*Arthrospira maxima*	129910	*Arthrobotrys oligospora*	13349	*Brassica rapa*	3711
*Arthrospira platensis*	118562	*Aspergillus terreus*	33178	*Castanea sativa*	21020
*Crocosphaera watsonii*	263511	*Beauveria bassiana*	176275	*Glycine max*	3847
*Cyanothece sp.*	43988	*Chaetomium thermophilum*	209285	*Hordeum vulgare*	4513
*Leptolyngbya boryana*	1184	*Claviceps purpurea*	5111	*Ipomoea nil*	35883
*Lyngbya aestuarii*	118322	*Colletotrichum higginsianum*	80884	*Lemna gibba*	4470
*Microcoleus chthonoplastes*	64178	*Cordyceps militaris*	73501	*Medicago truncatula*	3880
*Microcystis sp.*	1127	*Exophiala dermatitidis*	5970	*Mesembryanthemum crystallinum*	3544
*Moorea producens*	1155739	*Fusarium oxysporum*	5507	*Oryza sativa Japonica*	39947
*Oscillatoria sp.*	1159	*Gibberella zeae*	5518	*Phaseolus vulgaris*	3885
*Planktothrix rubescens*	59512	*Glomerella graminicola*	31870	*Populus nigra*	3691
		*Leptosphaeria maculans*	5022	*Ricinus communis*	3988
		*Metarhizium acridum*	92637	*Thellungiella halophila*	98038
		*Myceliophthora thermophila*	78579	*Triticum aestivum*	4565
		*Nectria haematococca*	140110	*Vitis vinifera*	29760
		*Paracoccidioides brasiliensis*	121759	*Zea mays*	4577
		*Podospora anserina*	5145		
		*Sordaria macrospora*	5147		
		*Talaromyces stipitatus*	28564		
		*Thielavia terrestris*	35720		
		*Trichoderma reesei*	51453		
		*Verticillium dahliae*	27337		

List of organisms from the six different phylum used in this work and their respective taxonomy IDs.

### Construction of Hidden Markov Models

In order to find remote similarity between model organisms and worm's clock proteins, the proteins involved in the circadian clock of the 5 better characterized chronobiology model organisms, *Drosophila melanogaster, Mus musculus, Synechococcus elongatus, Neurospora crassa* and *Arabidopsis thaliana* (table 8), were considered. Ortholog proteins to each of the core clock proteins of each model organisms were searched using the BlastP service of NCBI (http://blast.ncbi.nlm.nih.gov/), using the default cut off E-value. This search was restricted to the following phyla: 12 organisms with completely sequenced genomes belonging to C*hordata*, 7 for *Arthropoda*, 13 for Cyanobacteria, 24 for *Ascomycota*, and 18 for Tracheophyta (table 7). The set of positive hits was then aligned using the Muscle routine of MEGA v5.2 suite with default parameters [59]. Then, specific Hidden Markov Model profiles (HMM profiles) were generated for each alignment using the Hmmbuild routine from the HMMER3 software [28].

**Table 8:** List of circadian clock related proteins from the five model organisms used in this work.

Organism	Proteins
***Mus musculus***	PER1 - PER2 - PER3 - BMAL1 - BMAL2 – CLK - NPAS2 - CRY1 - CRY2 - RORA – RORB - NR1D1 - NR1D2 – DBP CSNK1a - CSNK1d - CSNK1e - CCRN4L - DEC-1 -DEC-2 FBXL-3 -PPARGC1a - MEL1A - MEL1B - OPN4 - PKC2 VIP - VPAC2 - PRDX-2 – WD-REP
***Drosophila melanogaster***	PER – TIM – CLK – CYC – CRY – VRI - PDP1E – CWO - CREB – DBT - CK2A - CK2B – SGG - PP1b - PP1-13C PP2a_MTS - PP2a_TWS - PP2a_WBT_A - PP2a_WBT_B PRMT-5 – SLMB - RH1 - RH5 - RH6 – NMO – NOCTE - NORPA – TO – PDF – PDFR – SLO – NA – IR – JET - CSN4 – PKA – EBONY – LRK - FER2 – JAFRAC - WDS
***Arabidopsis thaliana***	CCA1 - COP1 - DET1 – GI – LHY – LUX - PRR5 - PRR7 - PRR9 - TOC1
***Neurospora crassa***	FRH – FRQ - FWD-1 – VVD - WC-1 - WC-2
***Synechococcus elongatus***	KAI-A - KAI-B - KAI-C

### Search of the clock proteins in *Caenorhabditis elegans*

The HMM profiles described above were used as input to search for similar proteins in *Caenorhabditis elegans* using the HMMsearch routine from the same software. This resulted in 5 databases (one for each *Phylum*) containing the best hits for each HMM profile. Each protein of the collection of best hits resulted by the HMMsearch routine in *Caenorhabditis elegans* was further analyzed by InterProScan (http://www.ebi.ac.uk/Tools/pfa/iprscan/). Those proteins that contained the same domains as the query protein from each model organism were kept as *accepted hits* [103]. The default HMMsearch 3.0 inclusion threshold value for the full sequence length was used (0.01).

### Search of the clock orthologous proteins in the others worms

The *accepted hits* from *Caenorhabditis elegans* were then used as queries to perform local homology searches (BlastP software) against the IPDs of the other members of the *phylum Nematoda*. The default standalone blastP cut off E-value was used (1E^−10^). This resulted in a collection of the best hits for each clock protein from all the nematode proteomes. This list was again further refined by comparing the domains found in each protein and that of the accepted hit in *C. elegans* to obtain a list of those proteins conserved in the *phylum*. Finally the best hit for each worm was used as query against *Caenorhabditis elegans* IPD in order to find the reciprocity with an *ad hoc* reciprocal best hit (RBH) routine based in BlastP. The default standalone blastP cut off E-value was used (1E^−10^).

### Analysis of genetic circadian regulatory elements

All *C. elegans'* orthologs that corresponded to accepted clock prototypes were analyzed at the promoter level to search for known circadian regulatory elements with the next IUPAC syntaxis: E-box (CACGTG), D-box (TTATGYAA); RRE (WAWNTRGGTCA), GRE (ACANNNTGTTCT), PPRE (TGACCY), CREB (TGACGTMA), HSE (NGAANNGAANNTTCN), and CBP (TKGNGAAK) [78]–[80], [104]. Genomic sequences 3000 bp upstream of the ATG (translation start site) were downloaded for each putative clock gene with de WormMart tool [105]. Regulatory elements analysis was then performed with the jPREdictor v1.with default parameters [106].

### Phylogenetic analysis

A phylogenetic tree of the *phylum Nematoda* was built using Cytochrome B sequences, as previously described [107]. The sets of orthologous proteins were aligned by the Muscle routine from MEGA v5.2suite. The phylogenetic trees were constructed using the *neighbor-joining* method, the *Poisson* model for amino acid substitutions, a *Pairwise Deletion* for the *Gaps/Missing Data Treatment* and a *Gamma distributed* rate among sites was calculated for each alignment. The percentage of replicate trees where the *taxa* was grouped in the *bootstrap test* (1000 replicates) is shown at the side of each branch. The net distance between *taxa* was determined by the *Poisson* correction model.

## Supporting Information

Figure S1 **Phylogenetic tree of the core clock protein TIMELESS.** The phylogenetic trees were constructed using the *neighbor-joining* method, the *Poisson* model for amino acid substitutions, a *Pairwise Deletion* for the *Gaps/Missing Data Treatment* and a *Gamma distributed* rate among sites was calculated for each alignment. The percentage of replicate trees where the *taxa* was grouped in the *bootstrap test* (1000 replicates) is shown at the side of each branch. The net distance between *taxa* was determined by the *Poisson* correction model.
(TIF)

Figure S2 **Similar proteins are found among mammals, insects and nematodes.** The figure shows the seven *C. elegans'* proteins that are conserved among the clocks of mammals and insects, depicted in a: A) mammalian like clock model; and, B) insect (*Drosophila*) like clock model.
(TIF)

Table S1 **Conserved mammalian** ***C. elegans'*** **hits in all nematode species.** This table shows the 12 mammalian protein hits that are conserved in all other nematode species. *C. elegans'* best hit for each mammalian protein is used as a query to search each proteome. The database, protein hit, percentage of identity, alignment length, E value and bit score are detailed in the table.
(XLSX)

Table S2 **Conserved insect** ***C. elegans'*** **hits in all nematode species.** This table shows the 32 insect protein hits that are conserved in all other nematode species. *C. elegans'* best hit for each insect protein is used as a query to search each proteome. The database, protein hit, percentage of identity, alignment length, E value and bit score are detailed in the table.
(XLSX)

Table S3 **Promoter elements analysis.** This table shows the occurrence of putative circadian promoter elements in the genes of the conserved mammal/insect components, similar to insect components and similar to mammal components.
(XLSX)

Table S4 **Comparison between BlastP and HMM search strategies.** The table highlights the proteins that have been identified by the HMM search which were not found by the classical blastP approach.
(XLSX)

File S1 **HMM profiles used in this work and HMMsearch results.**
(ZIP)
