# Supplementary figures and images for: Potential Conservation of Circadian Clock Proteins in the phylum Nematoda as Revealed by Bioinformatic Searches
Source: PLoS One. 2014 Nov 14;9(11):e112871. doi: 10.1371/journal.pone.0112871 (PMC4232591; doi:10.1371/journal.pone.0112871)

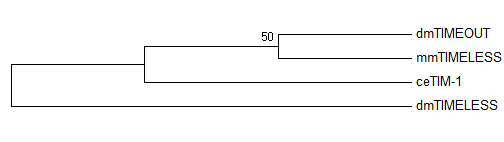

Supplement: Figure S1 — Phylogenetic tree of the core clock protein TIMELESS. The phylogenetic trees were constructed using the neighbor-joining method, the Poisson model for amino acid substitutions, a Pairwise Deletion for the Gaps/Missing Data Treatment and a Gamma distributed rate among sites was calculated for each alignment. The percentage of replicate trees where the taxa was grouped in the bootstrap test (1000 replicates) is shown at the side of each branch. The net distance between taxa was determined by the Poisson correction model. (TIF) [file pone.0112871.s001.tif]

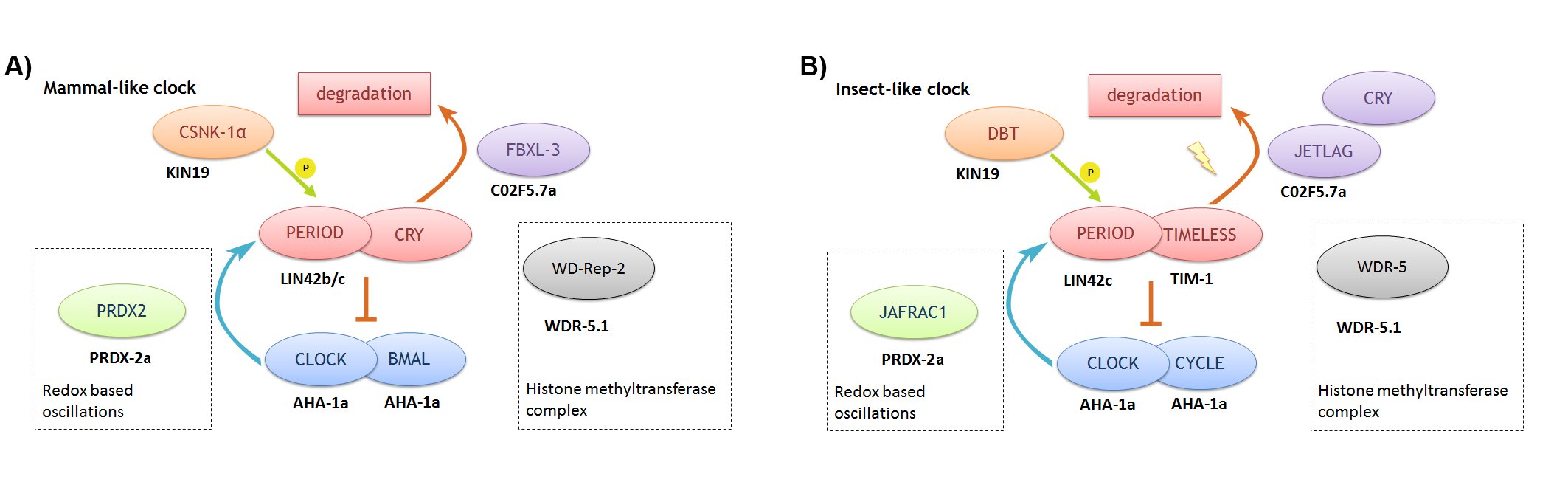

Supplement: Figure S2 — Similar proteins are found among mammals, insects and nematodes. The figure shows the seven C. elegans' proteins that are conserved among the clocks of mammals and insects, depicted in a: A) mammalian like clock model; and, B) insect (Drosophila) like clock model. (TIF) [file pone.0112871.s002.tif]
